# Spatio-temporal evolution analysis of land-use carbon effects driven by new quality productive forces: a case study of Hubei province

**DOI:** 10.1038/s41598-026-52454-2

**Published:** 2026-05-20

**Authors:** Xia Zhou, Yanwen Liu, Donghui Zhang, Senhao Liu, Yu Gao, Yurong Jiang

**Affiliations:** 1 School of Resources and Environment Science and Engineering, Hubei University of Science and Technology, Xianning, 437100 China; 2 Hubei Provincial Engineering Research Center for Intelligent Early Warning and Governance of Ecological Environment Changes in Small Watersheds, Xianning, 437100 China; 3 Research Center of Beidou + Industrial Development of Key Research Institute of Humanities and Social Sciences of Hubei Province, Xianning, 437100 China; 4 Institute of Remote Sensing Satellite, China Academy of Space Technology, Beijing, 100094 China; 5 School of Microelectronics and Communication Engineering, Chongqing University, Chongqing, 400044 China; 6 Aerospace Information Research Institute, Chinese Academy of Sciences (CAS), Beijing, 100101 China

**Keywords:** New quality productive forces (NQPF), Land use carbon emissions, Tapio decoupling, Spatial autocorrelation, Hubei Province, Education, Psychology, Psychology

## Abstract

This study investigated the spatiotemporal evolution and coupling relationship of land use carbon effects driven by new quality productive forces (NQPF) in Hubei Province, China, using remote sensing data (2000–2020) and socioeconomic statistics (2005–2020). An NQPF evaluation system comprising 19 indicators across three dimensions was constructed using a combined weighting method integrating entropy, coefficient of variation, CRITIC, and principal component analysis. The Tapio decoupling model and Moran’s I spatial autocorrelation were employed to examine the NQPF-carbon emission relationship. Results indicated that: (1) Net carbon emissions increased from 4118.38 × 104 t to 19,867.15 × 104 t (382.4% growth), with construction land contributing 98.1% of total carbon sources by 2020. (2) The NQPF index rose from 0.152 to 0.860 (465.8% growth), with the “new objects of labor” dimension receiving the highest weight (0.5921), corroborating the green development-oriented nature of NQPF. (3) All three periods exhibited “weak decoupling,” with elasticity coefficients declining from 0.740 to 0.261, indicating strengthening carbon constraints. (4) No significant spatial clustering was detected (|Z|< 1.96); riverside cities (Wuhan, Xiangyang, Yichang) formed “isolated high-emission centers” accounting for 54.2% of provincial emissions. This study pioneers the integration of NQPF theory into land use carbon research, providing scientific basis for regional low-carbon transition in central China.

## Introduction

In September 2023, General Secretary Xi Jinping first introduced the concept of “new quality productive forces (NQPF)” during his inspection tour of Heilongjiang Province, emphasizing the imperative to “actively cultivate strategic emerging industries, accelerate the formation of NQPF, and strengthen new drivers of development.” In January 2024, General Secretary Xi further elaborated that “developing NQPF constitutes both an intrinsic requirement and a central pillar of high-quality development, necessitating sustained efforts to promote innovation and accelerate their advancement.” As an advanced manifestation of productive forces, NQPF are fundamentally characterized by substantial improvements in total factor productivity. Driven primarily by scientific and technological innovation, they represent a fundamental departure from traditional economic growth patterns and conventional productivity development pathways^1^.

Land, serving as the fundamental carrier of productive activities, exerts a direct influence on productivity development through its utilization patterns. Since the reform and opening-up era, China’s rapid industrialization and urbanization have induced profound transformations in land use structure, with the expansion of construction land contributing substantially to increased carbon emissions^2–5^. According to the IPCC (2019) report, forestry, agriculture, and other land uses account for approximately 23% of total global anthropogenic greenhouse gas emissions^6^. Withinthe constraints imposed by the “dual carbon” targets, optimizing land use and reducing carbon emission intensity through the development of NQPF has emerged as a pressing practical challenge requiring urgent resolution. Achieving China’s carbon peak and carbon neutrality targets requires balancing economic growth paths with energy structure optimization. Previous studies have shown that China can achieve its carbon neutrality goal through economic growth rate adjustment and the development of a low-carbon energy structure^7^; further improving the quality of economic development could even advance carbon neutrality to before 2050^8^; and adjusting the energy consumption structure is a key pathway to achieving China’s CO₂ emissions peak^9^.

Hubei Province, as a strategically important province in central China, possesses abundant scientific and educational resources alongside a robust industrial foundation. Wuhan’s “Optics Valley” has attracted a substantial concentration of high-tech enterprises; Yichang has leveraged the Three Gorges Project to develop a clean energy industrial cluster; and Xiangyang is undergoing a transition toward intelligent manufacturing. Nevertheless, between 2000 and 2020, construction land in Hubei Province expanded from 5147 km^2^ to 8293 km^2^ (an increase of 61.12%), while land use carbon emissions escalated from 41.18 million tons to 198.67 million tons, representing a growth rate of 382.4%. This sustained increase in carbon sources underscores the inherent unsustainability of traditional development paradigms.

The academic community has conducted extensive investigations into the connotation, characteristics, and formation mechanisms of NQPF. Liu et al.^10^ conceptualized NQPF as productive capabilities that transcend traditional productive forces, emerging in alignment with the evolving demands of urban and rural residents. From a compositional perspective, NQPF encompass three dimensions: new laborers (knowledge-based, skilled, and innovative talents), new means of production (digital and intelligent tools), and new objects of labor (strategic emerging industries and future industries)^11,12^. Regarding measurement methodologies, existing studies predominantly employ objective weighting approaches, including the entropy method and principal component analysis^13^. However, current research has primarily focused on the spatiotemporal differentiation characteristics of NQPF, with relatively limited attention devoted to their coupling relationships with specific domains such as land use and carbon emissions^14–16^.

The accounting framework for land use carbon emissions has matured into a well-established research paradigm, encompassing methodologies such as the carbon emission coefficient method, life cycle assessment, and IPCC inventory approach^17–20^. The carbon emission coefficient method has gained widespread adoption owing to its superior data accessibility and computational simplicity. With respect to spatial patterns, scholars have employed spatial autocorrelation analysis, gravity center migration models, and standard deviational ellipse methods to elucidate the spatial evolution dynamics of carbon emissions^14^. Nevertheless, existing studies have predominantly treated land use carbon emissions as an isolated environmental issue, with insufficient integration of coupling analyses incorporating indicators of economic development quality and productivity levels.

The Tapio decoupling model has been extensively applied in quantitative assessments of the relationship between economic growth and resource-environmental pressures^21^. By calculating elasticity coefficients, this model categorizes the relationship between economic growth and environmental pressure into distinct types, including decoupling, coupling, and negative decoupling^22^. In the domain of carbon emissions research, scholars have utilized the Tapio model to analyze decoupling relationships among economic growth, energy consumption, industrial structure, and carbon emissions^19^. Incorporating NQPF development and carbon emissions within the Tapio decoupling framework enables a more comprehensive analysis of the interrelationship between carbon emissions and economic development.

This study employs Hubei Province as a case study, systematically analyzing the spatiotemporal evolution characteristics of land use carbon effects and their coupling relationships from the perspective of NQPF. The specific research objectives are as follows:

1. To calculate land use carbon emissions for Hubei Province and its 17 prefecture-level cities from 2000 to 2020, utilizing remote sensing monitoring data and the carbon emission coefficient method, and to analyze their spatiotemporal evolution characteristics.
2. To construct an evaluation indicator system for NQPF based on a three-dimensional framework comprising “new laborers – new means of labor – new objects of labor,” employing a combined weighting method (entropy, coefficient of variation, CRITIC, and principal component analysis) to quantify the development level of NQPF in Hubei Province from 2005 to 2020.
3. To apply the Tapio decoupling model to analyze the decoupling relationship and coordinated evolutionary trajectory between NQPF and land use carbon emissions.
4. To reveal the spatial clustering characteristics and spatial spillover effects of carbon emissions through Moran’s I spatial autocorrelation analysis.

## Materials and methods

### Overview of the study area

**Fig. 1:**
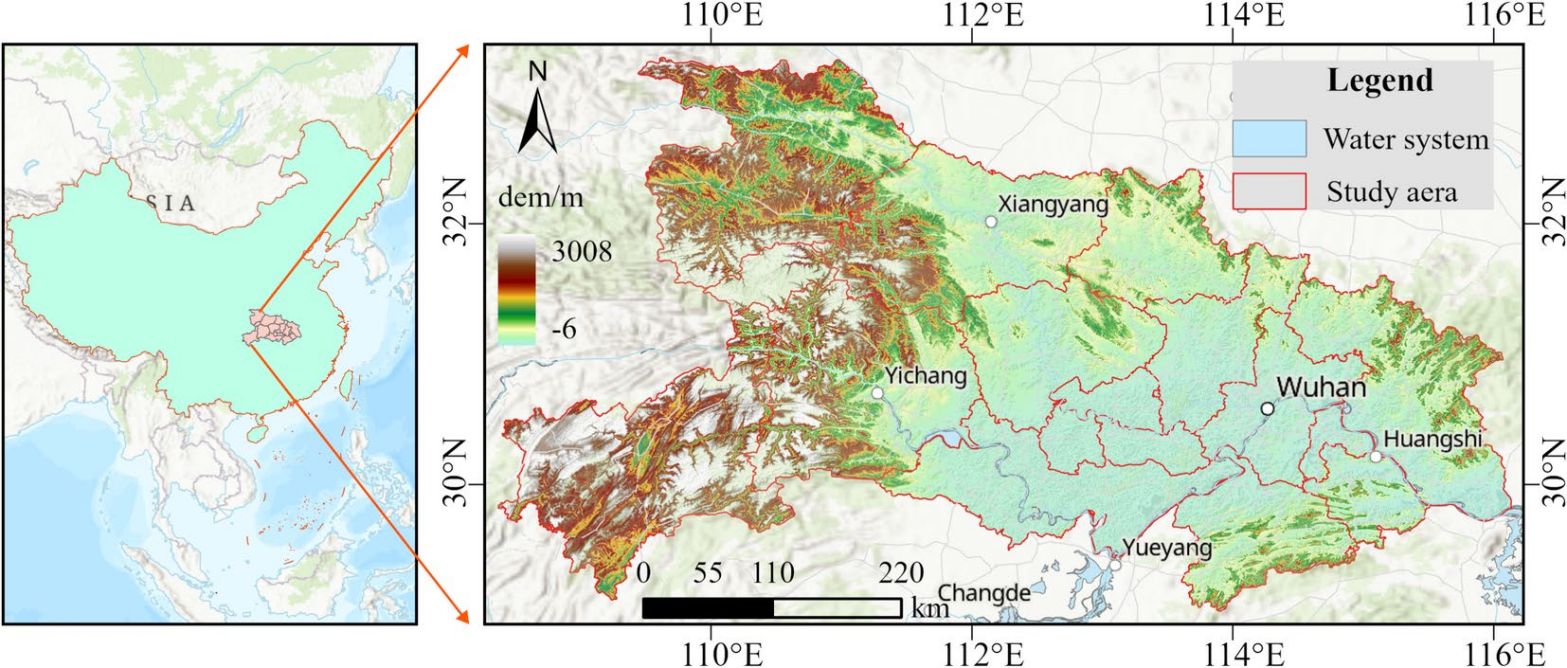
Geographical Overview of Hubei Province. Figures 1, 2 and 5 were generated using Python (version 3.10.12, https://www.python.org/, Python Software Foundation, USA) with the open‑source libraries Matplotlib (3.7.2, https://matplotlib.org/), Geopandas (0.14.1, https://geopandas.org/), and Cartopy (0.22.0,https://scitools.org.uk/cartopy/). The base map data are from Natural Earth Data and the Resource and Environment Science and Data Center, Chinese Academy of Sciences.

Hubei Province is situated in the middle reaches of the Yangtze River in central China, spanning geographical coordinates of 108°21′–116°07′E and 29°05′–33°20′N (Fig. 1). The province shares borders with Anhui to the east, Jiangxi and Hunan to the south, Chongqing to the west, and Henan and Shaanxi to the north, occupying a strategically vital position that connects eastern and western China while facilitating north–south communication. The province encompasses a total land area of approximately 186,000 km^2^ and administers 17 prefecture-level divisions, including the cities of Wuhan, Xiangyang, and Yichang, as well as autonomous prefectures and forest districts. The terrain is predominantly characterized by mountains, hills, and plains, with the Qinba and Wuling mountain ranges in the west, the Jianghan Plain in the central and eastern regions, and an overall topographic gradient descending from west to east in a stepped configuration^23^. Hubei Province experiences a subtropical monsoon climate, with mean annual temperatures ranging from 15 to 17 °C and annual precipitation of approximately 1,200 mm. The climate exhibits distinct seasonal variations, with concurrent periods of high temperature and abundant rainfall, providing favorable natural conditions for agricultural production and ecosystem development. As of 2020, Hubei Province had a permanent resident population of 57.75 million and a gross domestic product (GDP) of 4.34 trillion yuan. As a core province of the Yangtze River Economic Belt and a strategic pivot for the Rise of Central China initiative, Hubei Province confronts dual pressures of achieving high-quality economic development while controlling carbon emissions in its pursuit of NQPF and the “dual carbon” targets^24^. Consequently, investigating the spatiotemporal evolution patterns of land use carbon effects in this region holds significant theoretical and practical implications for optimizing regional carbon management strategies and facilitating green, low-carbon transformation.

### Data sources and preprocessing

The data sources and types employed in this study are summarized in Table 1. Land use data for Hubei Province across five temporal periods were derived from the China Land Use/Cover Change (CNLUCC) dataset produced by the Institute of Geographic Sciences and Natural Resources Research, Chinese Academy of Sciences. Based on the secondary classification system, the original data were reclassified into six categories: cultivated land, forestland, grassland, water bodies, construction land, and unused land. Administrative boundary data for Hubei Province were also obtained from the Resource and Environment Science and Data Center, Chinese Academy of Sciences. Data pertaining to energy consumption, economic development, and scientific and technological innovation used for constructing the NQPF indicators were compiled from the Hubei Statistical Yearbook and statistical yearbooks of individual prefecture-level cities within Hubei Province. All data were systematically collected and organized by category, with missing values in earlier years addressed through interpolation methods.

**Table 1:** Main data sources and data types.

Data type	Source	Format	Spatial resolution	Temporal resolution
Land use data	https://www.resdc.cn	Raster	30m	Year
Administrative boundary data	https://www.resdc.cn	Shape	–	Year
Hubei Provincial Statistical Yearbook	https://tjj.hubei.gov.cn	Table /Text	–	Year

### Methods

#### Land use carbon emission accounting

The carbon emission coefficient method is a technique employed to estimate greenhouse gas emissions. In this study, land use carbon emission accounting encompasses both direct and indirect calculation approaches. Direct carbon emissions refer to those resulting from changes in land types and land management practices; for cultivated land, forest, grassland, water bodies, and unused land, carbon emissions were calculated using the carbon emission coefficient method. Indirect carbon emissions are estimated based on statistical data related to human production activities; for construction land, carbon emissions were indirectly calculated through regional energy consumption data^25–27^.

### (1) Direct carbon emission calculation

Carbon emissions from cultivated land, forestland, grassland, water bodies, and unused land were directly calculated using the carbon emission coefficient method. The calculation formula is expressed as follows:

1 $${C}_{e}=\sum {e}_{n}=\sum_{n=1}^{5}{a}_{n}*{\varepsilon}_{n}$$

where ${C}_{e}$ represents the total carbon emissions from five land use types (cultivated land, forestland, grassland, water, and unused land); ${e}_{n}$ denotes the carbon emissions (or carbon sequestration) generated by the n-th land use type; ${a}_{n}$ represents the area of the n-th land type; and ${\varepsilon}_{n}$ is the carbon emission coefficient (expressed as positive values) or carbon sequestration coefficient (expressed as negative values) for the n-th land type.

Agricultural activities on cultivated land generate carbon emissions^23^; however, crops also sequester carbon through photosynthesis. Therefore, in determining the carbon emission coefficient for cultivated land, this study comprehensively considered both emission and sequestration processes. Preliminary calculations were conducted for carbon emissions from agricultural fertilizer application, machinery usage, and irrigation activities on cultivated land in Hubei Province from 2000 to 2020, alongside carbon sequestration by major crops. The results indicated that carbon emissions from cultivated land substantially exceeded carbon sequestration; consequently, cultivated land was classified as a carbon source in this study. The carbon emission coefficient for cultivated land was determined as the ratio of net carbon emissions to cultivated land area over the 2000–2020 period, yielding a value of 0.5963 tC/hm2, which is consistent with coefficients reported in previous studies^28^. Forestland, characterized by dense vegetation and active photosynthesis, is considered the largest carbon sink. Grassland constitutes another terrestrial ecosystem carbon sink with notable sequestration capacity. Water bodies possess certain carbon fixation capabilities due to phytoplankton and wetland characteristics. Unused land, despite containing scattered vegetation and microorganisms with relatively weak carbon absorption capacity, also exhibits limited carbon sink functionality^25^. The carbon emission coefficients for each land type were determined by synthesizing previous research findings with the specific conditions of Hubei Province and expert judgment. The coefficients for cultivated land, forestland, grassland, water bodies, and unused land were established as 0.5963 tC/hm2, − 0.6465 tC/hm2, − 0.0210 tC/hm2, − 0.2525 tC/hm2, and − 0.0051 tC/hm2, respectively^28–33^.

The carbon effect of cultivated land simultaneously involves two opposing processes: carbon emissions from agricultural activities and carbon sequestration from crop photosynthesis. Therefore, a single empirical coefficient cannot be directly applied. This study employs the “net carbon emission method” to estimate the cultivated land coefficient for Hubei Province. First, carbon emissions from cultivated land in Hubei Province from 2000 to 2020 were calculated, covering five emission sources: fertilizer application, agricultural film use, pesticide application, irrigation, and agricultural machinery use. Second, carbon sequestration by major crops (rice, wheat, corn, rapeseed, etc.) during the same period was estimated using a “yield → biomass → carbon uptake” conversion logic. Finally, the total net carbon emissions (emissions minus sequestration) were divided by the total cultivated land area to obtain an average netcarbon emission coefficient of 0.5963 tC/hm^2^. This positive value indicates that cultivated land in Hubei Province acts as a weak net carbon source, which is consistent with the province’s intensive agricultural production and high input intensity of agricultural materials. For forestland, grassland, water bodies, and unused land, the coefficients were directly adopted from ecologically recognized carbon sink intensity values, and their magnitude ordering (forestland > water bodies > grassland > unused land) aligns with the known carbon sink capacity ranking of different ecosystem types.

### (2) Indirect carbon emission calculation

Carbon emissions from construction land are typically estimated indirectly through energy consumption during land utilization^34,35^. During data collection, it was found that energy consumption statistics are available only at the provincial level, with comprehensive municipal-level data remaining incomplete. Consequently, using aggregate energy consumption data to estimate construction land carbon emissions would fail to capture regional variations among prefecture-level cities in Hubei Province.

Given that the output value of the secondary and tertiary industries is predominantly generated on construction land, energy consumption per unit of GDP serves as a comprehensive indicator of energy use intensity on construction land. Accordingly, this study calculated carbon emissions from construction land using energy consumption per unit of GDP and the combined GDP of secondary and tertiary industries for each of the 17 prefecture-level cities in Hubei Province. The calculation formula is as follows:

2 $${C}_{b}=GDP*{U}_{energy}*{\varepsilon}_{coal}$$

where ${C}_{b}$ represents the annual carbon emissions from construction land; GDP denotes the combined output value of secondary and tertiary industries (unit: 10,000 yuan); ${U}_{energy}$ represents the energy consumption per unit of GDP (unit: tons of standard coal per 10,000 yuan); and ${\varepsilon}_{coal}$ is the carbon emission coefficient for coal combustion, with ${\varepsilon}_{coal}=0.746 \mathrm{t}\mathrm{C}/\mathrm{t}\mathrm{o}\mathrm{n} \mathrm{o}\mathrm{f} \mathrm{s}\mathrm{t}\mathrm{a}\mathrm{n}\mathrm{d}\mathrm{a}\mathrm{r}\mathrm{d} \mathrm{c}\mathrm{o}\mathrm{a}\mathrm{l}$.

#### Construction of the NQPF index

Productive forces represent humanity’s capacity to utilize and transform nature for material production, encompassing three fundamental elements: laborers, objects of labor, and means of labor, which together constitute an interacting production system^8,9^. NQPF represent the developmental trajectory of advanced productive forces, characterized by innovation-driven growth that transcends traditional economic growth patterns and productivity development pathways. These forces exhibit attributes of high technology, high efficiency, and high quality, embodying an advanced form of productive forces aligned with the new development philosophy^10^.

### (1) Construction of the NQPF indicator system

Amid the ongoing technological revolution and industrial transformation, NQPF have emerged as an advanced form by superseding traditional productive forces, signifying a qualitative leap in productivity levels. This transition has given rise to new constituent elements of productive forces: new laborers, new objects of labor, and new means of labor. Drawing upon the conceptual framework of NQPF and the Marxist political economy analytical perspective on productive forces, and following a comprehensive literature review, this study constructed an evaluation indicator system for assessing the level of NQPF in Hubei Province. The system adheres to principles of objectivity, scientific rigor, and data availability, comprising three criterion layers, nine element layers, and nineteen indicators across three dimensions: new laborers, new means of labor, and new objects of labor (Table 2).

**Table 2:** Evaluation indicator system for NQPF in Hubei Province.

Target layer	Criteria layer	Element layer	Indicator layer	Unit	Code
New quality productive forces (NQPF)	New workers	Laborer quality	Number of university students enrollment	10,000 persons	A11
			Full-time equivalent of R&D personnel	Person-year	A12
		Employment structure	Proportion of urban employees in information transmission, software, and IT services	%	A21
			Proportion of urban employees in scientific research and technical services	%	A22
		Innovation activity	Expenditure on new product development in high-tech industries	10,000 CNY	A31
			Internal expenditure on R&D funds	10,000 CNY	A32
	New labor materials	Tools of labor	Length of railways in operation	km	B11
			Total length of highways	km	B12
			Length of long-distance optical fiber cable lines	10,000 km	B13
		Infrastructure	Mobile phone penetration rate	Sets per 100 persons	B21
			Number of broadband internet access users	10,000 households	B22
	New labor objects	New energy	New energy power generation	100 million kWh	C11
		New quality industries	Number of large and medium-sized enterprises	Unit	C21
			Number of enterprises with R&D activities	Unit	C22
			Value-added of high-tech industries	100 million CNY	C23
		Eco-environmental protection	Comprehensive utilization rate of general industrial solid waste	%	C31
			Ratio of industrial wastewater to total wastewater discharge	%	C32
		Sci-tech output	Number of patent applications accepted per 10,000 persons	Items/10,000 persons	C41
			Number of patents granted per 10,000 persons	Items/10,000 persons	C42

Specifically, new laborers constitute the principal agents in the production process. With the continuous advancement of science, technology, and society, knowledge-based, skilled, and innovative workers have become indispensable to scientific and technological innovation and serve as the primary drivers of NQPF development. Accordingly, this study measures the contribution of laborers to NQPF across three dimensions: laborer quality, employment structure, and innovation activity. Given that scientific and technological innovation constitutes the core element of NQPF, and that human capital represents the primary resource for such innovation, high-caliber talent forms the foundation for developing NQPF. Laborer quality is therefore characterized by the number of enrolled university students and the full-time equivalent of R&D personnel. Employment structure is measured by the proportions of urban employees in information transmission, software, and IT services, as well as in scientific research and technical services. Furthermore, financial investment serves as essential material support for scientific and technological activities while reflecting the innovation intensity of high-caliber talent. Hence, expenditure on new product development in high-tech industries and internal R&D expenditure were selected to characterize innovation activity.

New means of labor refer to the tools employed in production activities. Compared with traditional productive forces, NQPF involve emerging fields with high technological content, representing the evolution of traditional means of production under informatized and intelligent production conditions. In this study, new means of labor are evaluated through tools of labor and infrastructure, which constitute the foundational conditions for developing NQPF. Tools of labor are characterized by railway length in operation, total highway length, and long-distance optical fiber cable length. Infrastructure encompasses mobile phone penetration rate and the number of broadband internet subscribers.

New objects of labor are the materials upon which labor is applied through the means of labor. In response to the new round of technological revolution and industrial transformation, NQPF generate new industries and growth drivers through scientific and technological innovations such as artificial intelligence and the internet. Moreover, NQPF are inherently green productive forces; their development necessitates integration with the promotion of advanced green technologies and acceleration of the green transformation of development patterns. Consequently, this study measures new objects of labor through four dimensions: new energy, new quality industries, eco-environmental protection, and scientific and technological output. New energy power generation directly characterizes the new energy dimension. The number of large and medium-sized enterprises, the number of enterprises with R&D activities, and the value-added of high-tech industries characterize the degree of new quality industries. The comprehensive utilization rate of industrial solid waste and the ratio of industrial wastewater to total wastewater discharge characterize eco-environmental protection status. Patent applications accepted and patents granted per 10,000 persons characterize scientific and technological output.

### (2) Measurement methods for NQPF

Common methods employed by previous scholars for measuring NQPF include the entropy method, entropy-TOPSIS method, principal component analysis (PCA), coefficient of variation method, CRITIC method, and BP neural network models^10,36^. To ensure objective and comprehensive measurement of NQPF, this study proposes a combined weighting approach that integrates four commonly used objective weighting methods to enhance the robustness of indicator weight estimation.

The entropy method determines weights based on information entropy theory through the information content of indicators. The calculation formulas are:

3 $${e}_{j}=-\frac{1}{\mathrm{ln}n}{\sum}_{i=1}^{n}{p}_{ij}\mathrm{ln}{p}_{ij}$$

4 $${d}_{j}=1-{e}_{j}$$

5 $${w}_{j}^{Entory}=\frac{{d}_{j}}{{\sum}_{j=1}^{m}{d}_{j}}$$

where ${e}_{j}$ is the information entropy of the j-th indicator, ${d}_{j}$ is the information utility value, and ${p}_{ij}$ is the standardized indicator value. A smaller entropy value indicates greater information content and thus higher weight.

The coefficient of variation method determines weights based on the relative variation of indicators:

6 $$C{V}_{j}=\frac{{\upsigma}_{j}}{{\upmu}_{j}}$$

7 $${w}_{j}^{CoV}=\frac{C{V}_{j}}{{\sum}_{j=1}^{m}C{V}_{j}}$$

where ${\upsigma}_{j}$ and ${\upmu}_{j}$ are the standard deviation and mean of the j-th indicator, respectively. A larger coefficient of variation corresponds to higher weight.

The CRITIC method comprehensively considers indicator contrast intensity and conflict:

8 $${C}_{j}={\sum}_{k=1}^{m}\left(1-{r}_{jk}\right)$$

9 $${I}_{j}={\sigma}_{j}\times {C}_{j}$$

10 $${w}_{j}^{CRITIC}=\frac{{I}_{j}}{{\sum}_{j=1}^{m}{I}_{j}}$$

where ${r}_{jk}$ is the correlation coefficient between indicators, ${C}_{j}$ is the conflict index, and ${I}_{j}$ is the information quantity.

PCA extracts principal components and calculates weights based on factor loadings:

11 $${w}_{j}^{PCA}=\frac{{\sum}_{k=1}^{p}\left|{l}_{jk}\right|\times {\uplambda}_{k}}{{\sum}_{j=1}^{m}{\sum}_{k=1}^{p}\left|{l}_{jk}\right|\times {\uplambda}_{k}}$$

where ${l}_{jk}$ is the loading of the j-th indicator on the k-th principal component, and${\uplambda}_{k}$ is the variance contribution rate of the k-th principal component.

The combined weighting method balances the advantages of each approach, with final weights calculated as the arithmetic mean:

12 $${w}_{j}^{Combination}=\frac{1}{4}\left({w}_{j}^{Entory}+{w}_{j}^{CoV}+{w}_{j}^{CRITIC}+{w}_{j}^{PCA}\right)$$

This method effectively reduces biases inherent in single methods and enhances the reliability of weight estimation.

This study combines four objective weighting methods: the entropy method, the coefficient of variation (COV) method, the CRITIC method, and principal component analysis (PCA). These four methods measure indicator importance from four complementary perspectives: indicator dispersion (entropy method), relative volatility (COV method), contrast intensity and conflict (CRITIC method), and global variance structure (PCA). Combining them avoids the systematic bias inherent in any single method. Regarding the integration strategy, equal averaging (arithmetic mean) is adopted instead of accuracy-based weighted averaging for the following main reasons: (1) equal averaging is the most commonly used parameter-free ensemble strategy in combination evaluation, offering good reproducibility and robustness against overfitting; (2) as shown in Fig. 6a, the weight distributions of the four methods are relatively close, so equal averaging does not cause significant distortion; and (3) introducing additional subjective weights would reduce the objectivity of the evaluation. Therefore, this study uses equal averaging as shown in Eq. (12) to calculate the final combined weights.

## Integrated analysis of NQPF and carbon emissions

### Tapio decoupling index

To elucidate the temporal coupling relationship between NQPF development and land use carbon emissions in Hubei Province, this study employs the Tapio decoupling model^37^ for quantitative assessment of their dynamic association. By measuring the relative rates of change between economic growth and environmental pressure^22^, this model effectively identifies the response patterns of carbon emissions during the development of NQPF, providing a quantitative basis for evaluating whether regional development has achieved green transformation.

The Tapio decoupling index is calculated as follows:

13 $$\varepsilon =\frac{\Delta CE/C{E}_{0}}{\Delta NQPF/NQP{F}_{0}}=\frac{\left(C{E}_{t}-C{E}_{t-1}\right)/C{E}_{t-1}}{\left(NQP{F}_{t}-NQP{F}_{t-1}\right)/NQP{F}_{t-1}}$$

where $\varepsilon$ denotes the decoupling elasticity coefficient; $C{E}_{t}$ and $C{E}_{t-1}$ represent net land use carbon emissions in periods t and t + 1, respectively; $NQP{F}_{t}$ and $NQP{F}_{t-1}$ represent the composite index of NQPF in periods t and t + 1, respectively; $\Delta CE$ and $\Delta NQPF$ represent the changes in carbon emissions and the NQPF index, respectively.

Based on Tapio’s classification criteria and considering the development scenarios of NQPF during corresponding periods (where $\Delta NQPF$>0), this study categorizes decoupling states into the following types:

1. *Strong Decoupling*: $\varepsilon <0$, indicating that carbon emissions decrease in absolute terms while NQPF increase, representing the ideal state of green development.
2. *Weak Decoupling*: $0\le \varepsilon \le 0.8$, indicating that carbon emissions continue to grow but at a rate significantly slower than that of NQPF, suggesting improving development quality and a critical transitional phase toward strong decoupling.
3. *Expansive Coupling*: $0.8\le \varepsilon \le 1.2$, indicating that carbon emissions and NQPF grow synchronously with a tight coupling relationship, signifying that effective decoupling has not yet been achieved.
4. *Expansive Negative Decoupling*: $\varepsilon \ge 1.2$, indicating that the growth rate of carbon emissions exceeds that of NQPF, reflecting an extensive development pattern with intensifying environmental pressure.

This study calculates the decoupling index for three periods (2005–2010, 2010–2015, and 2015–2020) to analyze the evolutionary trends of land use carbon emissions during the development of NQPF in Hubei Province. The analysis identifies temporal variation characteristics in decoupling intensity and explores the driving mechanisms underlying transitions between decoupling states across different development stages, thereby providing scientific evidence for evaluating the low-carbon effects of NQPF.

### Moran’s I spatial autocorrelation analysis

Moran’s I index is a classical statistic for measuring spatial autocorrelation of geographic phenomena, first proposed by Moran in 1950 and subsequently widely applied in spatial pattern analysis across fields such as regional economics and environmental science^14^. This study employs the global Moran’s I index to quantify the spatial clustering characteristics of carbon emissions across the 17 prefecture-level cities in Hubei Province. The calculation formula is:

14 $$I=\frac{n}{{S}_{0}}\times \frac{{\sum}_{i=1}^{n}{\sum}_{j=1}^{n}{w}_{ij}\left({x}_{i}-\overline{x }\right)\left({x}_{j}-\overline{x }\right)}{{\sum}_{i=1}^{n}{\left({x}_{i}-\overline{x }\right)}^{2}}$$

where n is the number of spatial units (n = 17); ${x}_{i}$ is the net carbon emissions of city i; $\overline{x }$ is the global mean; ${w}_{ij}$ is an element of the spatial weight matrix; and ${S}_{0}={\sum}_{i=1}^{n}{\sum}_{j=1}^{n}{w}_{ij}$ is the sum of all weights.

The spatial weight matrix is constructed using the inverse distance method to reflect the decay effect of geographical proximity. The distance ${d}_{ij}$ between cities is calculated using the Haversine spherical distance formula:

15 $${d}_{ij}=2R\cdot \mathrm{arcsin}\left(\sqrt{{\mathrm{sin}}^{2}\left(\frac{\Delta\upphi }{2}\right)+\mathrm{cos}\left({\upphi}_{i}\right)\mathrm{cos}\left({\upphi}_{j}\right){\mathrm{sin}}^{2}\left(\frac{\Delta\uplambda }{2}\right)}\right)$$

where $R=6371km$ is the Earth’s radius, $\upphi$ and $\uplambda$ denote latitude and longitude in radians, respectively. The weight matrix is defined as ${w}_{ij}=1/{d}_{ij}$ (when $i \ne j$), ${w}_{ii}=0$, ensuring that spatially proximate cities receive higher association weights.

Based on the weight matrix, the spatial lag variable for each city is calculated, representing the weighted average emission level of surrounding cities:

16 $${SL}_{i}=\frac{{\sum}_{j=1}^{n}{w}_{ij}\cdot {x}_{j}}{{\sum}_{j=1}^{n}{w}_{ij}}$$

Moran’s I ranges from − 1 to 1. Under the randomization hypothesis, the theoretical expected value is $E\left(I\right)=-1/\left(n-1\right)$. Values of $I>E\left(I\right)$ indicate positive spatial autocorrelation, $I<E\left(I\right)$ indicates negative autocorrelation, and $I\approx E\left(I\right)$ suggests random distribution. Statistical significance is assessed by constructing a Z-statistic:

17 $$Z\left(I\right)=\frac{I-E\left(I\right)}{\sqrt{Var\left(I\right)}}$$

where $Var(I)$ is the asymptotic variance. The null hypothesis of random distribution is rejected when $\mid Z\mid >1.96$(at $\alpha =0.05$).

To visually illustrate local spatial association patterns, this study constructs a Moran scatter plot with standardized carbon emissions $Z\left({x}_{i}\right)$ on the horizontal axis and standardized spatial lag $Z\left({SL}_{i}\right)$ on the vertical axis. Cities are classified into four spatial pattern types: HH (high-high clustering), LL (low-low clustering), HL (high-value outliers), and LH (low values surrounded by high values). The slope of the fitted line approximates the Moran’s I value, providing a visual representation of global spatial autocorrelation intensity.

## Results and analysis

### Spatiotemporal evolution of land use

**Fig. 2:**
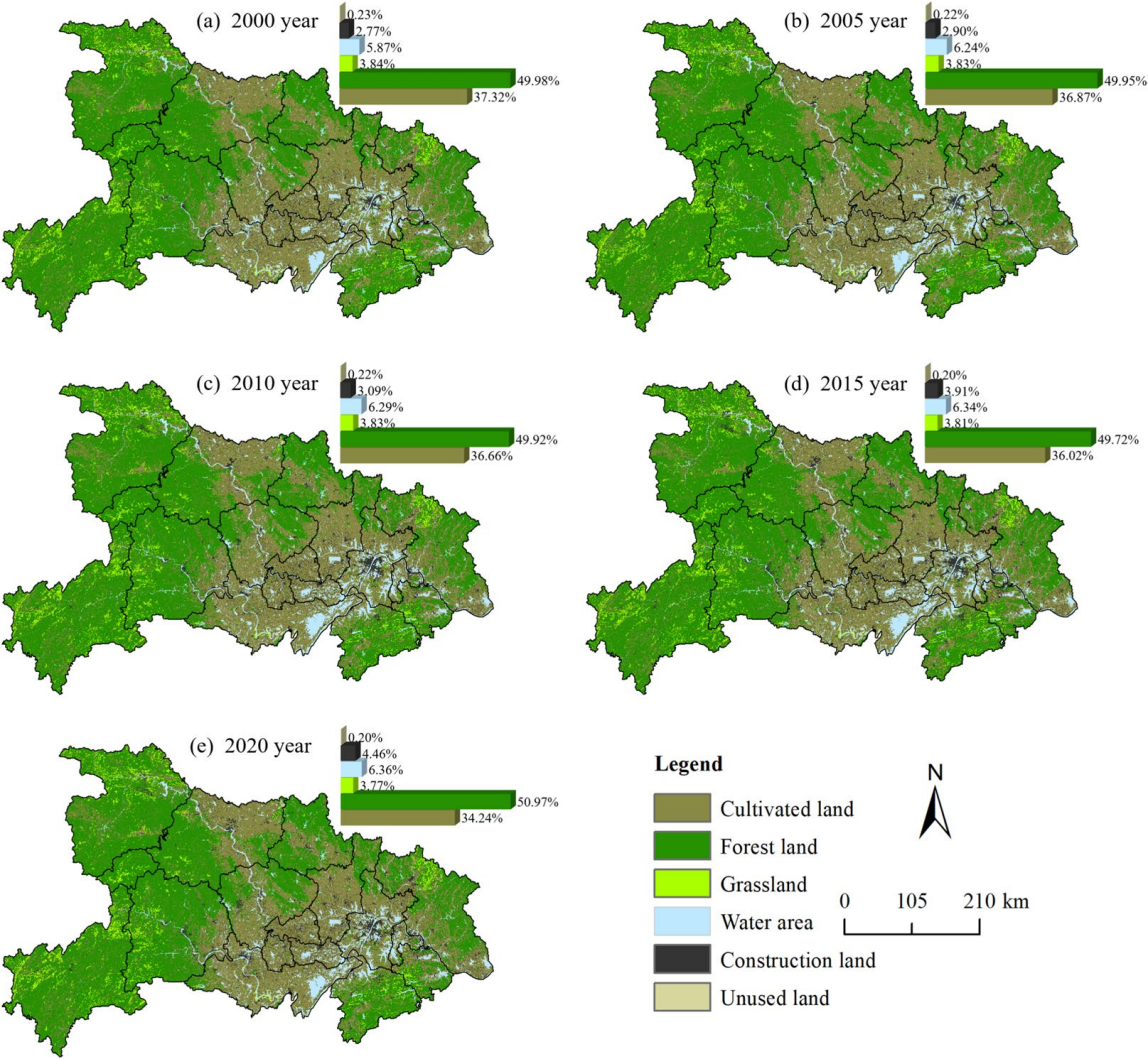
Land use change in Hubei Province at five-year intervals (2000–2020). Figures 1, 2 and 5 were generated using Python (version 3.10.12, https://www.python.org/, Python Software Foundation, USA) with the open‑source libraries Matplotlib (3.7.2, https://matplotlib.org/), Geopandas (0.14.1, https://geopandas.org/), and Cartopy (0.22.0, https://scitools.org.uk/cartopy/). The base map data are from Natural Earth Data and the Resource and Environment Science and Data Center, Chinese Academy of Sciences.

Based on remote sensing data across five periods from 2000 to 2020 (Fig. 2; Table 3), land use structure in Hubei Province underwent substantial transformation. In terms of overall proportions (Fig. 2), forestland consistently occupied the largest share (49.72%–50.97%), followed by cultivated land (34.24%–37.32%) and water bodies (5.87%–6.36%). Construction land increased from 2.77% to 4.46%, while grassland and unused land together accounted for less than 4%.

**Table 3:** Land use changes of various types in Hubei Province from 2000 to 2020 (Unit: km^2^).

Years	2000	2005	2010	2015	2020
Cultivated land	69,430	68,598	68,197	67,016	63,705
Forestland	92,971	92,916	92,861	92,493	94,822
Grassland	7138	7119	7121	7089	7022
Water area	10,919	11,600	11,708	11,786	11,829
Construction land	5147	5396	5743	7277	8293
Unused land	430	406	405	374	364

**Fig. 3:**
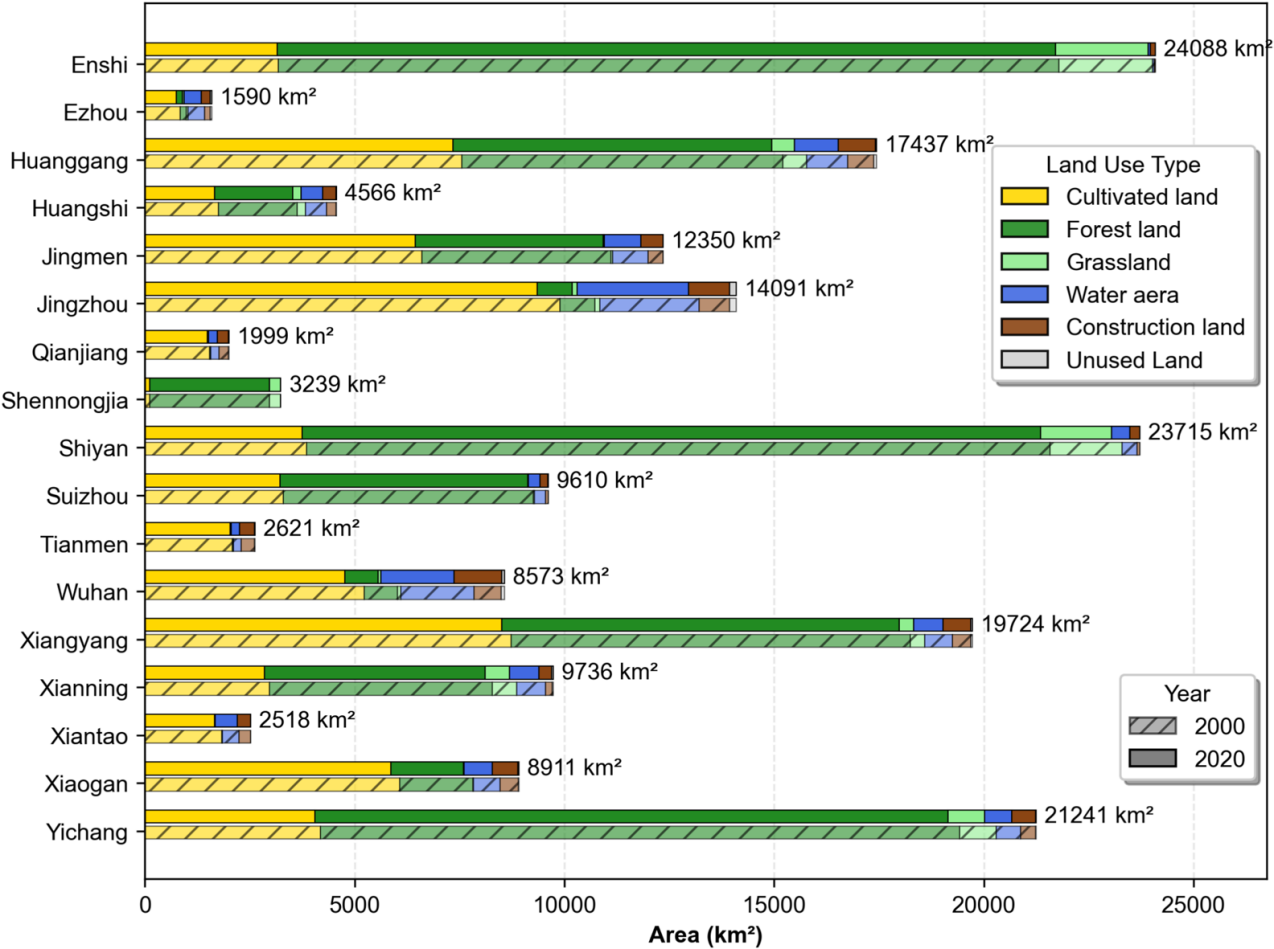
Land use composition in variouscities and prefectures of Hubei Province.

**Fig. 4:**
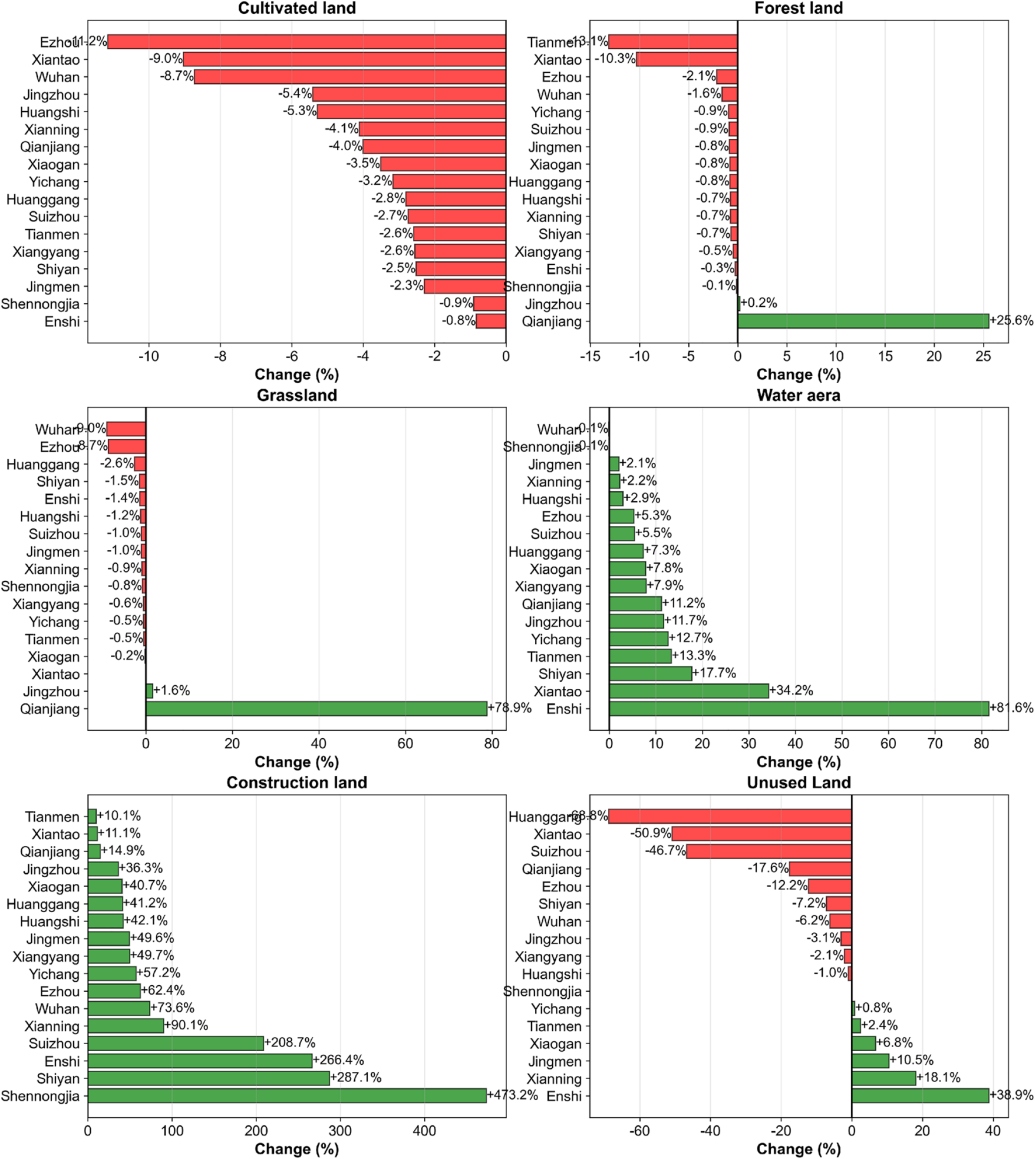
Changes in land use types in various cities and prefectures of Hubei Province from 2000 to 2020 (relative to the area in 2000).

The expansion of construction land was the most pronounced, with area increasing from 5147 km^2^ to 8293 km^2^, representing a net increase of 3146 km^2^ and a growth rate of 61.13% (Table 3). As illustrated in Fig. 3, construction land (shown in brown) expanded noticeably across all prefecture-level cities between 2000 and 2020, with particularly marked increases in Wuhan, Xiangyang, Yichang, and Huanggang. Regarding the rate of construction land change (Fig. 4), Shennongjia Forest District exhibited the highest growth rate (+ 473.2%), albeit from a small base (3239 km^2^). Shiyan, Enshi, and Suizhou also recorded growth rates exceeding 200%. Wuhan increased by 73.6%, Xiangyang by 49.7%, and Yichang by 57.2%, with these three cities collectively accounting for nearly 60% of the province’s net increase in construction land. As shown in Table 3, construction land expansion displayed an accelerating trend, with a net increase of approximately 1016 km^2^ during 2015–2020, substantially exceeding the totals of the preceding three five-year periods.

Cultivated land exhibited continuous decline, decreasing from 69,430 km^2^ to 63,705 km^2^, representing a net loss of 5725 km^2^ (− 8.24%) (Table 3). As shown in Fig. 3, cultivated land proportion decreased from 37.32% in 2000 (indicated by yellow hatching) to 34.24% in 2020 (solid yellow), with spatial distribution concentrated primarily in the Jianghan Plain and the northern Hubei uplands. According to Fig. 4, Ezhou experienced the most severe cultivated land loss (− 11.2%), followed by Xiantao (− 9.0%) and Wuhan (− 8.7%). These areas are predominantly located in the core zone of the Jianghan Plain, where urbanization pressure is considerable.

Forestland area fluctuated marginally, increasing slightly from 92,971 km^2^ to 94,822 km^2^, representing a net gain of 1851 km^2^ (+ 1.99%). As shown in Fig. 2, forestland (dark green) is primarily distributed in the western Hubei mountainous region (Enshi, Shennongjia) and the southeastern Hubei mountainous region (Huanggang), with its proportion remaining stable between 49 and 51%. According to Fig. 4, Qianjiang exhibited the highest rate of forestland area change (+ 25.6%), primarily attributable to the fact that the majority of Qianjiang’s land consists of cultivated land. Combined with Table 3, the net increase of approximately 2000 km^2^ in forestland during 2015–2020 indicates the significant effectiveness of the Grain-for-Green Program.

Water body area increased from 10,919 km^2^ to 11,829 km^2^, representing a net gain of 910 km^2^ (+ 8.33%). As illustrated in Fig. 2, the proportion of water bodies (light blue) increased from 5.87 to 6.36%. According to Fig. 4, Enshi exhibited the largest change in water body area (+ 81.6%), primarily attributable to the construction of cascade hydropower stations along the Qingjiang River. Water bodies in Xiantao increased by 34.2%, associated with the Central Route of the South-to-North Water Diversion Project. However, water body areas decreased in Huanggang, Xianning, and other cities in the Jianghan Plain, reflecting pressure from lake shrinkage.

### Spatiotemporal evolution of land use carbon accounting

#### Overall carbon emission trends in Hubei province

Based on the land use carbon emission accounting model, the spatiotemporal evolution characteristics of land use carbon emissions in Hubei Province from 2000 to 2020 were calculated (Table 4). In terms of overall trends, net land use carbon emissions in Hubei Province increased from 4118.38 × 104 t in 2000 to 19,867.15 × 104 t in 2020, representing a growth rate of 382.4%.

**Table 4:** Carbon emissions from different land use types in Hubei Province, 2000–2020 (× 10^4^ t).

Years	2000	2005	2010	2015	2020
Cultivated land	415.72	410.7	398.2	393.07	399.45
Forestland	− 600.16	− 599.43	− 599.51	− 597.64	− 596.31
Grassland	− 1.49	− 1.48	− 1.46	− 1.45	− 1.47
Water area	− 27.31	− 29.08	− 31.22	− 31.27	− 29.52
Construction land	4331.64	6909.06	13,623.42	17,255.92	20,095.02
Unused land	− 0.02	− 0.02	− 0.02	− 0.02	− 0.02
Carbon source	4747.36	7319.76	14,021.62	17,648.99	20,494.48
Carbon sink	− 628.98	− 630.01	− 632.21	− 630.38	− 627.32
Net carbon emissions	4118.38	6689.74	13,389.41	17,018.61	19,867.15

Regarding carbon source composition, construction land constituted the dominant contributor to carbon emissions. Carbon emissionsfrom construction land increased from 4331.64 × 104 t in 2000 to 20,095.02 × 104 t in 2020, representing a net increase of 15,763.38 × 104 t and a growth rate of 363.9%. The proportion of construction land emissions relative to total carbon sources rose from 91.2% in 2000 to 98.1% in 2020, indicating that energy consumption and construction activities associated with industrialization and urbanization were the principal drivers of carbon emission growth. Carbon emissions from cultivated land remained relatively stable, declining marginally from 415.72 × 104 t to 399.45 × 104 t (− 4.0%), with its share of total carbon sources decreasing from 8.8 to 1.9%. This reduction is primarily attributable to the controlled application of chemical fertilizers and pesticides, as well as the transformation of agricultural production practices.

With respect to carbon sink composition, forestland served as the predominant carbon sink, with sequestration volumes remaining stable between 596 × 104 t and 600 × 104 t, accounting for 94.7%–95.0% of total carbon sinks. Carbon sequestration by water bodies increased from 27.31 × 104 t to 29.52 × 104 t (+ 8.1%), comprising 4.3%–4.7% of total carbon sinks. Carbon sequestration by grassland and unused land was negligible (both below 1.5 × 104 t), contributing minimally to overall carbon sink capacity. Total carbon sinks declined marginally from 628.98 × 104 t in 2000 to 627.32 × 104 t in 2020 (− 0.3%), indicating that the carbon sink function of ecosystems in Hubei Province remained relatively stable.

Regarding temporal evolution, net carbon emissions exhibited a three-stage pattern characterized by “rapid growth–sustained growth–decelerated growth.” The period from 2000 to 2005 represented a rapid growth phase, during which net carbon emissions increased from 4118.38 × 104 t to 6 689.74 × 104 t, with an average annual growth rate of 10.2%. The period from 2005 to 2010 constituted a sustained growth phase, with net carbon emissions rising to 13,389.41 × 104 t at an average annual growth rate of 14.9%. From 2010 to 2020, growth decelerated, with emissions increasing from 13,389.41 × 104 t to 19,867.15 × 104 t at an average annual growth rate of 4.0%. This evolutionary pattern is closely associated with industrialization processes, energy consumption structure, and environmental policy adjustments in Hubei Province.

#### Carbon emission variations across prefecture-level cities in Hubei province

**Fig. 5:**
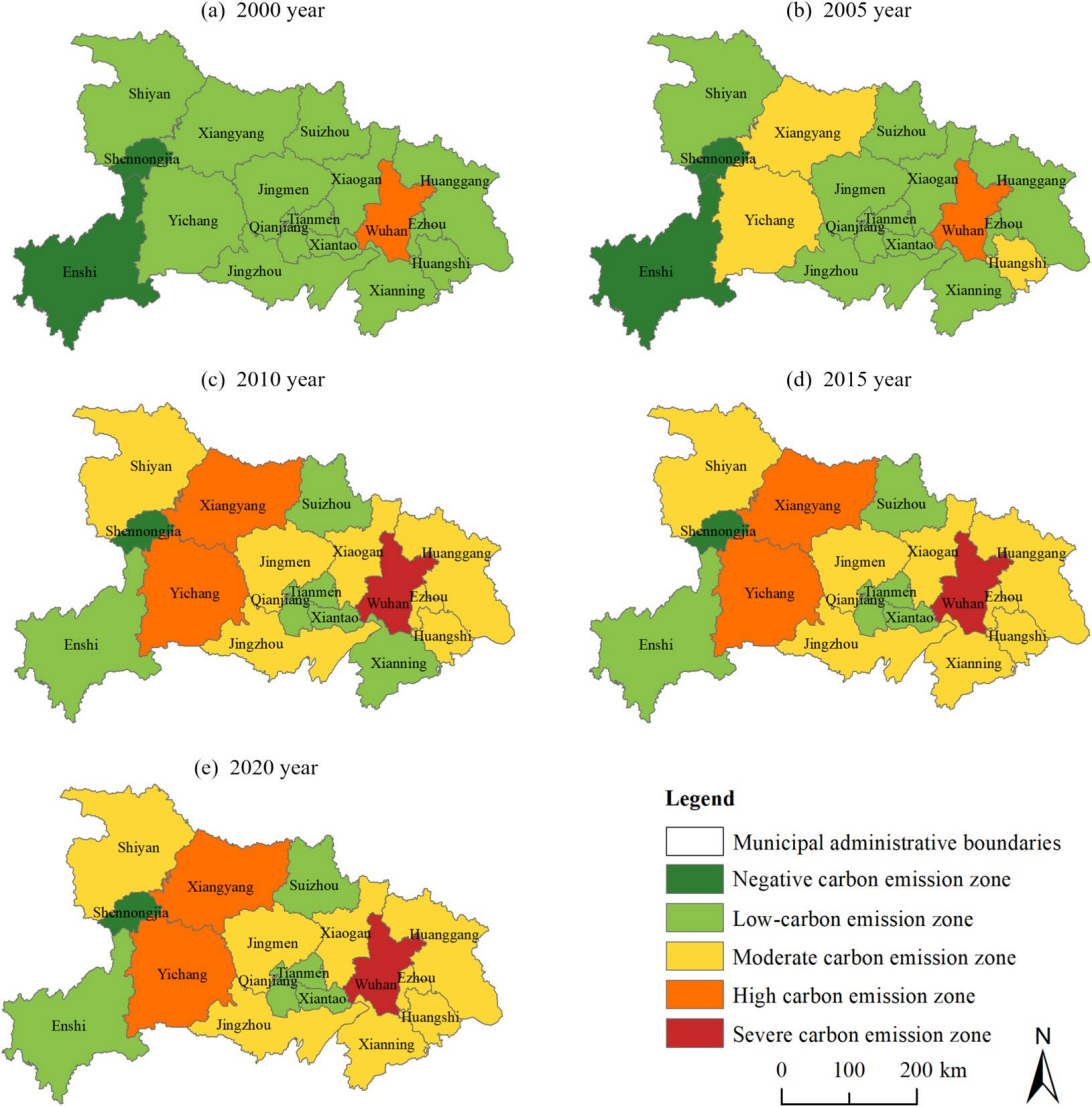
Spatiotemporal distribution of carbon emissions across prefecture-level cities in Hubei Province. Figures 1, 2 and 5 were generated using Python (version 3.10.12, https://www.python.org/, Python Software Foundation, USA) with the open‑source libraries Matplotlib (3.7.2, https://matplotlib.org/), Geopandas (0.14.1, https://geopandas.org/), and Cartopy (0.22.0, https://scitools.org.uk/cartopy/). The base map data are from Natural Earth Data and the Resource and Environment Science and Data Center, Chinese Academy of Sciences.

Based on carbon emission accounting at the prefecture-level city scale, the 17 prefecture-level cities in Hubei Province exhibited pronounced spatial differentiation (Fig. 5; Table 5). Using the K-means clustering method, municipal carbon emissions were classified into five categories: negative carbon emission zone (NC, [− ∞, 0]), low carbon emission zone (LC, [0.01, 426.00] × 104 t), moderate carbon emission zone (MC, [426.01, 1,181.23] × 104 t), high carbon emission zone (HC, [1181.24, 2361.57] × 104 t), and very high carbon emission zone (SC, [2361.58, 6249.87] × 104 t).

**Table 5:** Classification of land use carbon emissions in Hubei Province and city counts by category (× 10^4^ t).

Years	Clustering interval	Number of cities				
		2000	2005	2010	2015	2020
NC emission zone	[-∞, 0]	2	2	1	1	1
LC emission zone	[0.01, 426.00]	14	11	6	5	5
MC emission zone	[426.01, 1181.23]	0	3	7	8	8
HC emission zone	[1181.24, 2361.57]	1	1	2	2	2
SC emission zone	[2361.58, 6249.87]	0	0	1	1	1

Regarding the spatial evolution pattern (Fig. 5), carbon emissions in Hubei Province in 2000 displayed a “single core with one zone” configuration: Wuhan was the sole high carbon emission zone (orange), Shennongjia and Enshi constituted negative carbon emission zones (dark green), and the remaining 15 prefecture-level cities were classified as low carbon emission zones (light green). By 2005, the spatial pattern of carbon emissions began to shift, with Xiangyang, Yichang, and Huangshi transitioning to moderate carbon emission zones (yellow). By 2010, spatial diffusion of carbon emissions became evident: Wuhan transitioned to the very high carbon emission zone (red),Xiangyang and Yichang were elevated to high carbon emission zones (orange), and seven cities—Shiyan, Jingzhou, Huanggang, Xiaogan, Suizhou, Xianning, and Ezhou—progressed to moderate carbon emission zones, with only Shennongjia maintaining negative carbon emissions. During 2015–2020, the spatial pattern stabilized, with Wuhan remaining in the very high carbon emission zone, Xiangyang and Yichang consistently classified as high carbon emission zones, and the number of cities in the moderate carbon emission zone stabilizing at eight.

In terms of changes in city counts (Table 5), the number of cities in the negative carbon emission zone decreased from two in 2000 (Enshi, Shennongjia) to one in 2020 (Shennongjia). Cities in the low carbon emission zone declined sharply from 14 to 5 (Enshi, Suizhou, Qianjiang, Tianmen, Xiantao). Cities in the moderate carbon emission zone increased from 0 to 8, those in the high carbon emission zone increased from 1 to 2 (Xiangyang, Yichang), and cities in the very high carbon emission zone increased from 0 to 1 (Wuhan). These changes indicate that carbon emissions in Hubei Province exhibited dual trends of “carbon intensification” and “spatial agglomeration.”

Examining the evolution of representative cities, Wuhan, as the provincial capital and economic center, experienced a surge in net carbon emissions from 1,581.24 × 104 t in 2000 to 6,249.87 × 104 t in 2020, representing an increase of 295.2% and accounting for 31.5% of the province’s total net carbon emissions, thereby establishing itself as the “super core” of carbon emissions in Hubei Province. Xiangyang and Yichang, as regional hub cities, reached carbon emissions of 2,361.07 × 104 t and 2,144.26 × 104 t in 2020, accounting for 11.9% and 10.8% of the provincial total, respectively. Collectively, these three cities accounted for 54.2% of the province’s carbon emissions, forming a triangular high-emission configuration. In contrast, Shennongjia, designated as an ecological function zone, recorded carbon emissions of − 2.56 × 104 t in 2020, continuously serving as a carbon sink and functioning as an ecological security barrier for Hubei Province.

Regarding the underlying causes of the spatial pattern, the formation of the Wuhan–Xiangyang–Yichang high carbon emission axis is closely associated with industrial distribution along the Yangtze and Han Rivers, transportation advantages, and urbanization levels. The western Hubei mountainous region (Enshi, Shennongjia) and the southeastern Hubei mountainous region (parts of Huanggang, Xianning) maintained low-carbon characteristics owing to high forest coverage and low industrialization levels. Cities in the Jianghan Plain (Jingzhou, Jingmen, Xiaogan, Huangshi, and others) experienced gradual increases in carbon emissions, progressing to the moderate emission zone as a result of industrial development and agricultural modernization.

### Construction and temporal trends of NQPF indicators in Hubei Province

#### Combined weighting method for measuring NQPF

**Fig. 6:**
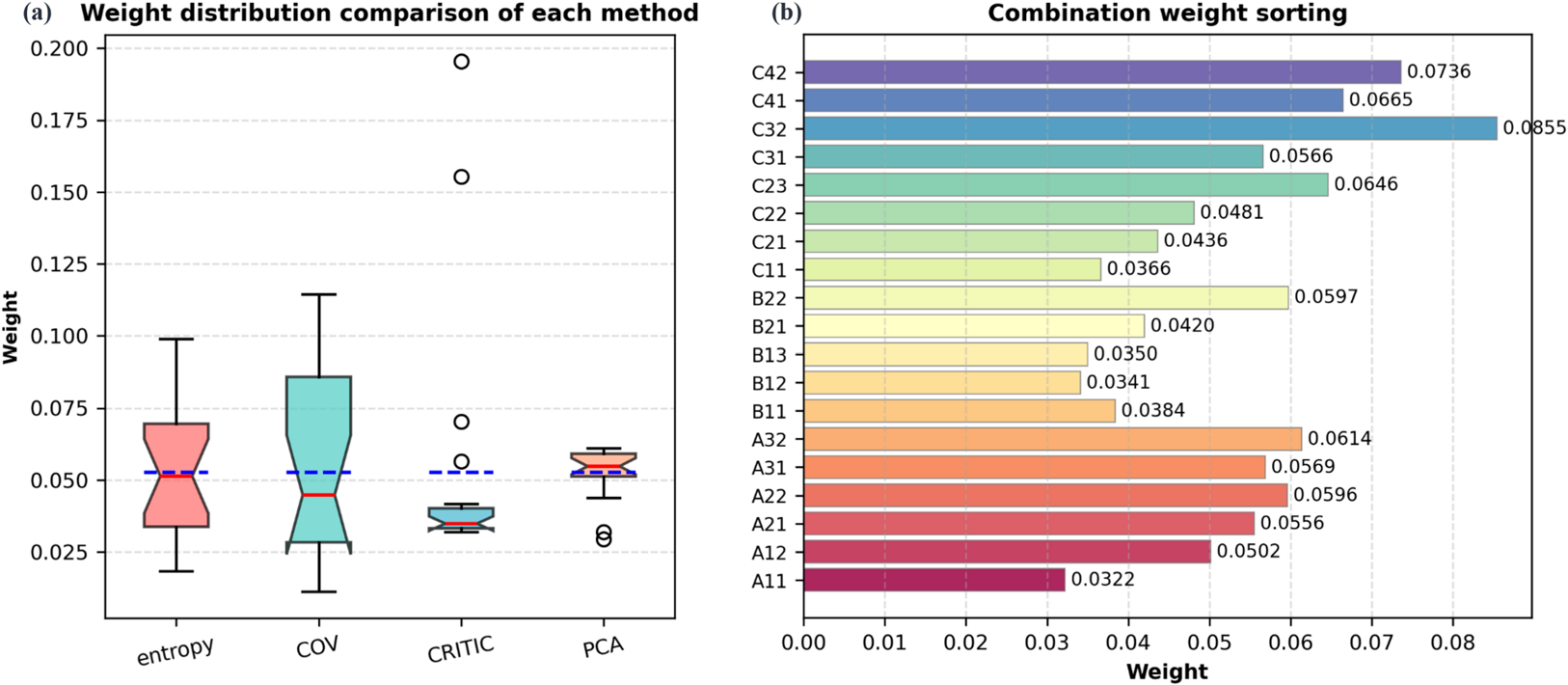
Weight distribution of NQPF indicators: (**a**) four individual methods; (**b**) combined weighting method.

Based on the three-dimensional framework of “new laborers–new means of labor–new objects of labor,” an evaluation system for NQPF comprising 19 indicators was constructed. To enhance the scientific rigor and robustness of weight assignment, this study integrated four objective weighting methods: the entropy method, the coefficient of variation (COV) method, the CRITIC method, and PCA, with combined weights derived through arithmetic averaging (Fig. 6; Table 6).

**Table 6:** Indicator weights for NQPF calculated using the combined weighting method.

Indicators code	Entropy	COV	CRITIC	PCA	Combined
A11	0.0275	0.0152	0.0336	0.0524	0.0322
A12	0.0560	0.0577	0.0321	0.0548	0.0502
A21	**0.0782**	0.0449	0.0387	**0.0604**	0.0556
A22	**0.0990**	0.0251	**0.0704**	0.0439	0.0596
A31	0.0514	0.0850	0.0320	0.0592	0.0569
A32	0.0676	**0.0869**	0.0319	**0.0592**	**0.0614**
B11	0.0321	0.0314	0.0366	0.0534	0.0384
B12	0.0183	0.0295	0.0374	0.0512	0.0341
B13	0.0355	0.0158	**0.0417**	0.0470	0.0350
B21	0.0360	0.0468	0.0339	0.0513	0.0420
B22	0.0607	**0.0868**	0.0320	**0.0595**	0.0597
C11	0.0293	0.0272	0.0368	0.0531	0.0366
C21	0.0465	0.0420	**0.0565**	0.0295	0.0436
C22	0.0542	0.0475	0.0338	0.0571	0.0481
C23	**0.0716**	**0.0953**	0.0329	0.0587	**0.0646**
C31	0.0278	0.0113	**0.1554**	0.0320	0.0566
C32	0.0493	0.0409	**0.1955**	0.0561	**0.0855**
C41	**0.0753**	**0.0964**	0.0340	**0.0601**	**0.0665**
C42	**0.0837**	**0.1145**	0.0349	**0.0612**	**0.0736**

The formatting (bold and underline) is used to identify the five indicators with the highest weights obtained from each of the five weight calculation methods (Entropy, COV, CRITIC, PCA, Combined).

Regarding the weight distribution characteristics of the four methods (Fig. 6a), the entropy and COV methods yielded relatively dispersed weight distributions, with ranges of 0.025–0.100 and 0.025–0.115, respectively, and larger standard deviations, indicating heightened sensitivity to indicator variability. In contrast, the CRITIC method and PCA produced more concentrated weight distributions, predominantly within the ranges of 0.030–0.040 and 0.050–0.060, with smaller standard deviations, reflecting consideration of inter-indicator correlations. The median weights across the four methods were 0.051, 0.045, 0.035, and 0.055, respectively. With the exception of two indicators assigned disproportionately high weights by the CRITIC method, the overall distributions were relatively comparable, providing a sound basis for combined weighting.

Concerning the combined weighting results (Fig. 6b; Table 6), the combined weights of the 19 indicators ranged from 0.0322 to 0.0855, with moderate variation that avoided the extreme weight allocations potentially arising from any single method. The five indicators with the highest weights were: C42 (scientific and technological output—patent grants per 10,000 persons, 0.0736), C41 (scientific and technological output—patent applications per 10,000 persons, 0.0665), C32 (ecological and environmental protection—ratio of industrial wastewater to total wastewater discharge, 0.0855), C23 (new quality industries—value added of high-tech industries, 0.0646), and A32 (laborer innovation activity—internal R&D expenditure, 0.0614). These five indicators are concentrated primarily within the “new objects of labor” dimension (C32, C23) and the “new laborers” dimension (C42, C41), suggesting that the intensity of scientific and technological innovation and green sustainable development constitute the core elements of NQPF.

Examining the weight distribution across the three dimensions, the “new laborers” dimension (A11–A32) received a combined weight of 0.1892, the “new means of labor” dimension (B11–B22) received 0.2187, and the “new objects of labor” dimension (C11–C42) received the highest combined weight of 0.5921. This result corroborates the essential attribute of NQPF as being “green development-oriented,” indicating that industrial structure optimization, resource utilization efficiency improvement, and ecological environmental protection occupy dominant positions in the development of NQPF.

Regarding individual indicator weights, within the “new laborers” dimension, A32 (laborer innovation activity—internal R&D expenditure, 0.0614) and A22 (laborer innovation activity—proportion of scientific research and technical services personnel in urban employment, 0.0596) received relatively high weights, indicating that talent quality serves as a critical foundation for NQPF. Within the “new means of labor” dimension, B22 (infrastructure—number of broadband internet subscribers, 0.0597) ranked second, reflecting the importance of digital infrastructure and innovation entities. Within the “new objects of labor” dimension, C32 (ecological and environmental protection—ratio of industrial wastewater to total wastewater discharge, 0.0855) received the highest weight, highlighting the pivotal role of green environmental development and energy structure transformation in NQPF advancement.

#### Temporal evolution characteristics of NQPF index

**Fig. 7:**
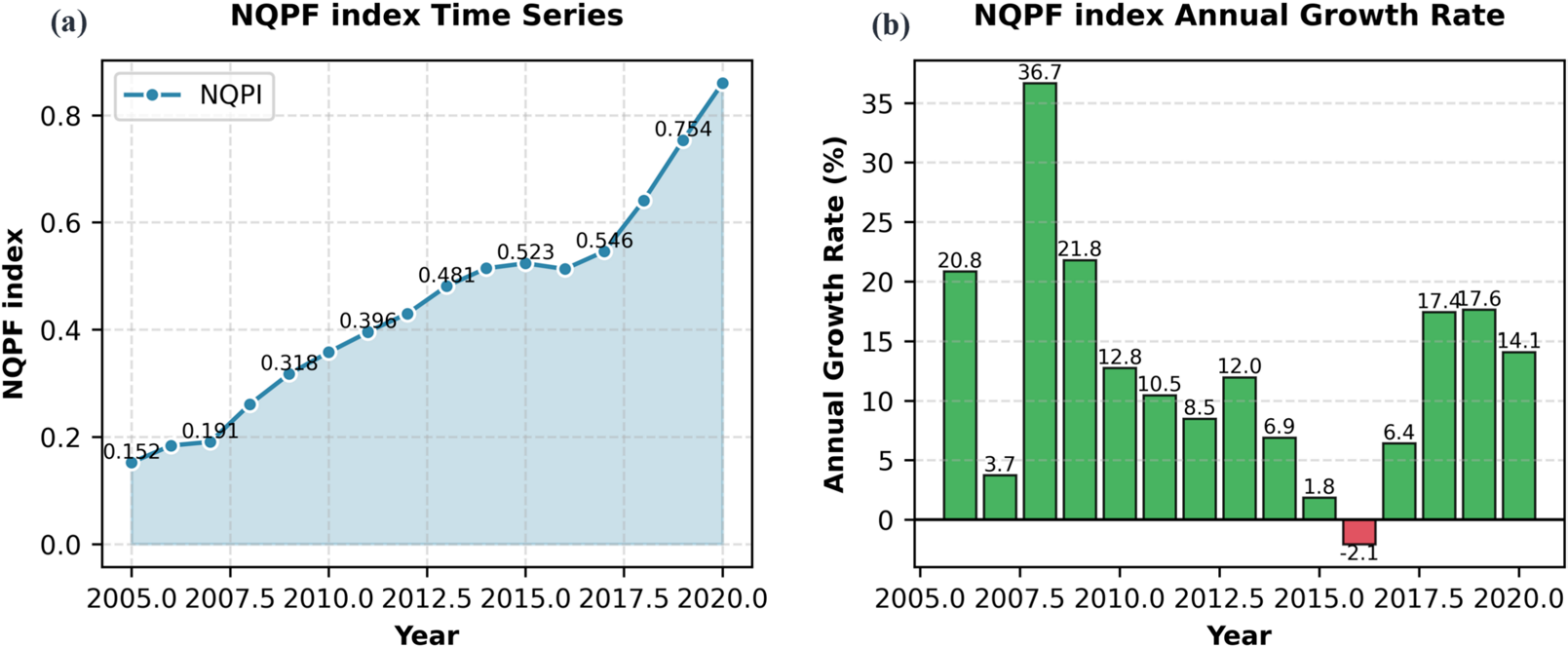
(**a**) Temporal evolution of the NQPF index; (**b**) Annual growth rate of the NQPF index.

The temporal evolution characteristics of the NQPF index (NQPF index) in Hubei Province from 2005 to 2020 were calculated using the combined weighting method (Fig. 7). In terms of overall trends (Fig. 7a), the NQPF index in Hubei Provinceincreased continuously from 0.152 in 2005 to 0.860 in 2020, representing a cumulative increase of 0.708, a total growth rate of 465.8%, and an average annual growth rate of 12.26%, demonstrating a pronounced upward trajectory.

Regarding growth phase characteristics (Fig. 7b), NQPF development exhibited a “W-shaped” fluctuating growth pattern, which can be delineated into four stages:

Stage I (2005–2007): Initiation phase. The NQPF index increased from 0.152 to 0.191 at a moderate pace. During this period, Hubei Province was undergoing rapid traditional industrialization, with strategic emerging industries yet to achieve scale, and insufficient accumulation of talent, technology, and capital.

Stage II (2007–2011): Rapid growth phase. The NQPF index rose substantially from 0.191 to 0.396, with an average annual growth rate of 20.0% (36.7% in 2007–2008, 21.8% in 2008–2009, and 12.8% in 2009–2010). This period benefited from the implementation of the national strategy for “enhancing indigenous innovation capacity.” The Wuhan Metropolitan Circle was designated as a national comprehensive reform pilot zone for a “resource-conserving and environmentally friendly society” in 2007, during which policy dividends from scientific and technological innovation and industrial transformation were substantially released in Hubei Province.

Stage III (2011–2016): Stable growth phase. The NQPF index increased from 0.396 to 0.513, with the average annual growth rate declining to 5.32%. Growth decelerated markedly in 2015 (1.8%), with brief negative growth observed in 2016 (− 2.1%). This slowdown was primarily attributable to the national economic transition to the “new normal,” during which policies such as capacity reduction in traditional industries and supply-side structural reform exerted short-term impacts on certain indicators.

Stage IV (2016–2020): Accelerated advancement phase. The NQPF index increased rapidly from 0.513 in 2016 to 0.860, with the average annual growth rate rebounding to 13.78% (6.4% in 2016–2017, 17.4% in 2017–2018, 17.6% in 2018–2019, and 14.1% in 2019–2020). During this period, Hubei Province implemented the innovation-driven development strategy in depth. The Wuhan Metropolitan Area Development Plan received approval (the 13th Five-Year Plan for Promoting the Rise of Central China in 2016 supported the construction of Wuhan as a nationally influential science and technology innovation center). The “Optics Valley” brand of the East Lake High-Tech Development Zone continued to gain prominence, and strategic emerging industries including integrated circuits, new-generation displays, and biopharmaceuticals experienced rapid growth, marking the transition of NQPF into a high-quality development stage.

Concerning annual fluctuation characteristics, two growth peaks occurred during 2007–2008 (36.7%) and 2017–2019 (17.4%–17.6%), corresponding to the policy-driven effects of the “resource-conserving and environmentally friendly society” pilot zone designation in 2007 and the accelerated emergence of strategic emerging industries, respectively. The brief decline during 2015–2016 (from 1.8 to − 2.1%) reflected transitional difficulties during economic restructuring; however, the subsequent rapid recovery demonstrated the strong resilience of NQPF development in Hubei Province. Overall, NQPF development in Hubei Province followed an evolutionary trajectory characterized by “policy-driven initiation–fluctuating growth–accelerated advancement.” The index value of 0.860 in 2020 indicates that Hubei Province achieved substantial progress in innovation-driven development, industrial upgrading, and green transformation.

### Coupling analysis of land use carbon effects driven by NQPF in Hubei Province

#### Tapio decoupling analysis of NQPF and carbon emissions

Based on the Tapio decoupling model, the decoupling relationship between carbon emissions (CE) and new quality productive forces (NQPF) development in Hubei Province from 2005 to 2020 was calculated (Table 7). Regarding overall evolutionary trends, carbon emissions and NQPF development in Hubei Province exhibited “weak decoupling” status across all three periods; however, the degree of decoupling displayed pronounced phase-specific characteristics. The decoupling elasticity coefficient decreased progressively from 0.740 to 0.261, indicating a strengthening constraining effect of NQPF on carbon emissions growth.

**Table 7:** Decoupling index calculation results by period.

Period	CE growth rate (%)	NQPF growth rate (%)	ε	Decoupling type
2005–2010	+ 100.15	+ 135.25	0.740	Weak decoupling
2010–2015	+ 27.11	+ 46.13	0.588	Weak decoupling
2015–2020	+ 16.74	+ 64.25	0.261	Weak decoupling

Examining decoupling characteristics by period, 2005–2010 represented a “low-degree decoupling phase,” with carbon emission growth rate reaching 100.15%, NQPF growth rate at 135.25%, and decoupling elasticity coefficient ε of 0.740. Although decoupling between carbon emissions and NQPF was achieved during this period, carbon emissions growth remained relatively rapid, equivalent to 74% of NQPF growth. The primary reasons include: Hubei Province was undergoing rapid industrialization, with substantial expansion of construction land (as shown in Table 4, carbon emissions from construction land increased from 6909.06 × 104 t to 13,623.42 × 104 t between 2005 and 2010, representing a 97.2% increase); traditional energy-intensive industries constituted a high proportion of the economy; the energy consumption structure remained coal-dominant; and green low-carbon technologies had not yet been deployed at scale.

The period 2010–2015 represented a “moderate-degree decoupling phase,” with carbon emission growth rate declining to 27.11%, NQPF growth rate at 46.13%, and decoupling elasticity coefficient decreasing to 0.588. Carbon emission growth decelerated substantially during this period, with marked improvement in decoupling status, primarily attributable to industrial restructuring and the implementation of energy conservation and emission reduction policies in Hubei Province. Notably, the 18th National Congress of the Communist Party of China in 2012 introduced the “ecological civilization construction” strategy, prompting Hubei Province to accelerate the phase-out of obsolete production capacity and promote cleaner production and circular economy development, thereby achieving a degree of control over carbon emission growth.

The period 2015–2020 represented a “high-intensity decoupling phase,” with carbon emission growth rate furtherdeclining to 16.74%, NQPF growth rate at 64.25%, and decoupling elasticity coefficient decreasing substantially to 0.261. During this period, NQPF growth (64.25%) was 3.84 times that of carbon emission growth (16.74%), indicating improving development quality and a critical transitional stage toward strong decoupling. The primary contributing factors include: first, the rapid emergence of strategic emerging industries, with increasing shares of low-carbon sectors such as optoelectronics and biopharmaceuticals; second, enhanced green technology innovation capacity and increased renewable energy consumption; third, substantial improvements in energy utilization efficiency with continuous declines in carbon emission intensity per unit of GDP; and fourth, the deepening advancement of ecological civilization construction, with carbon peaking and carbon neutrality targets guiding green transformation.

In summary, the relationship between carbon emissions and NQPF development in Hubei Province evolved from “low-degree decoupling” toward “high-intensity decoupling,” with the decoupling elasticity coefficient exhibiting a continuous declining trend (0.740 → 0.588 → 0.261). This indicates that NQPF development is effectively promoting the transformation of economic growth patterns in Hubei Province from high-carbon to low-carbon modes, with continuously improving green development quality.

#### Moran’s I spatial autocorrelation analysis

**Fig. 8:**
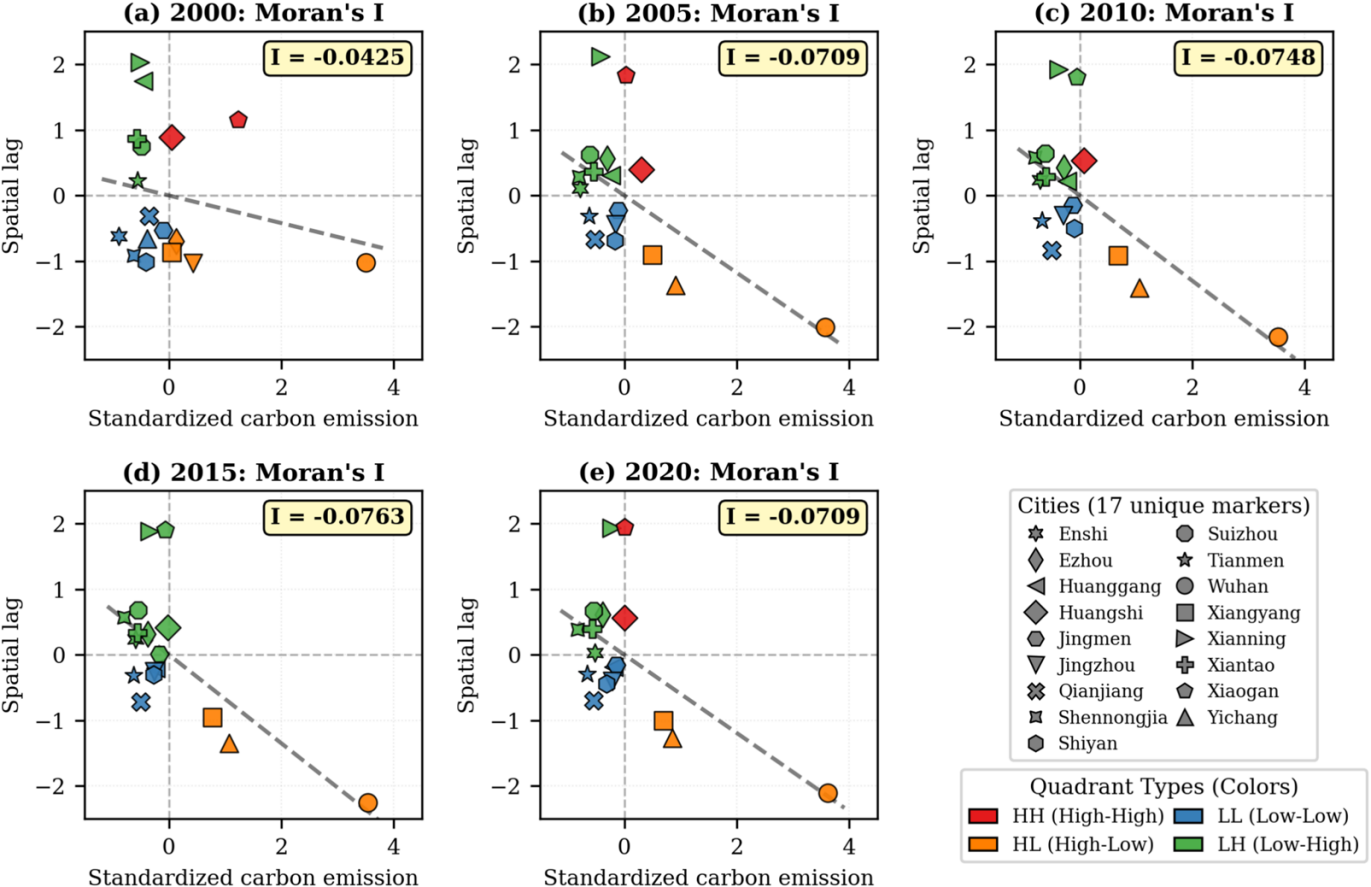
Moran scatter plots for prefecture-level cities, 2000–2020.

Based on Moran’s I index, the spatial autocorrelation characteristics of carbon emissions across the 17 prefecture-level cities in Hubei Province from 2000 to 2020 were analyzed (Table 8; Fig. 8). Regarding global spatial autocorrelation, Moran’s I values for all five years were negative and proximate to the expected value E(I) =  − 0.0625, specifically − 0.0425 (2000), − 0.0709 (2005), − 0.0748 (2010), − 0.0763 (2015), and − 0.0709 (2020). Deviation values did not pass significance testing (|Z|< 1.96), indicating that carbon emissions in Hubei Province at the prefecture-level city scale exhibited no significant spatial clustering characteristics overall, displaying a spatially random distribution pattern.

**Table 8:** Spatial autocorrelation characteristics of carbon emissions in Hubei Province.

Year	Moran’s I	E(I)	Deviation	Autocorrelation type
2000	− 0.0425	− 0.0625	+ 0.0200	Weak positive/random
2005	− 0.0709	− 0.0625	− 0.0084	Weak negative/random
2010	− 0.0748	− 0.0625	− 0.0123	Weak negative
2015	− 0.0763	− 0.0625	− 0.0138	Weak negative
2020	− 0.0709	− 0.0625	− 0.0084	Weak negative/random

Concerning temporal evolutionary trends, Moran’s I index exhibited a U-shaped pattern characterized by initial decline followed by subsequent increase. From 2000 to 2015, Moran’s I decreased continuously (− 0.0425 →  − 0.0763), with progressively strengthening negative correlation, indicating increasing spatial heterogeneity in carbon emissions and intensifying spatial differentiation between high-emission and low-emission areas. During this period, carbon emissions grew rapidly in central cities such as Wuhan, Xiangyang, and Yichang, while ecological function zones including Enshi and Shennongjia maintained low or negative emissions, resulting in expanding spatial disparities. From 2015 to 2020, Moran’s I increased slightly (− 0.0763 →  − 0.0709), suggesting a trend toward relative stabilization in the spatial distribution of carbon emissions, potentially associated with Hubei Province’s promotion of coordinated regional development and implementation of differentiated carbon reduction policies.

Regarding local spatial autocorrelation patterns (Fig. 8), the spatial configuration of carbon emissions in Hubei Province exhibited differentiated characteristics of “high-carbon isolates along rivers–low-carbon clustering in inland areas.” First, the HH quadrant (high-high clustering) contained very few cities with fluctuating membership (only Huangshi and Xiaogan appeared in the HH quadrant in certain years during 2000–2020), failing to form stable high-carbon clusters. Second, the LL quadrant (low-low clustering) contracted from 7 cities in 2000 to 5–6 cities during 2015–2020, forming a stable low-carbon cluster centered on Jingmen, Jingzhou, Qianjiang, Shiyan, and Tianmen. Third, the HL quadrant (high-low outlier) remained highly stable, with Wuhan and Xiangyang consistently positioned in the HL quadrant throughout 2000–2020, while Yichang became stably positioned in the HL quadrant from 2005 onward. These cities emerged as typical high-carbon isolates, with carbon emissions substantially exceeding those of surrounding areas. Fourth, the LH quadrant (low–high outlier) contained the largest number of cities. Xianning, Xiantao, and Suizhou remained consistently in the LH quadrant throughout 2000–2020, while Enshi, Ezhou, and Shennongjia entered and remained stable in the LH quadrant from 2005, forming a low-carbon depression pattern.

Overall, the spatial pattern of carbon emissions in Hubei Province exhibited pronounced differentiated characteristics of “high-carbon isolates along rivers–low-carbon clustering in inland areas”: riverside cities including Wuhan, Xiangyang, and Yichang formed stable high-carbon isolates (HL); inland cities including Jingmen, Jingzhou, Qianjiang, and Shiyan formed low-carbon clusters (LL); transitional cities including Xianning, Ezhou, and Suizhou formed low-carbon depressions (LH). Spatial heterogeneity was significant but clustering was not pronounced, with carbon emissions in individual cities primarily driven by their own development patterns and limited spatial spillover effects.

It should be noted that a non-significant global Moran’s I does not imply a complete absence of spatial structure; rather, it indicates the absence of systematic high-high (HH) or low-low (LL) clustering. In spatial analysis, global non-significance and local significance can coexist. This commonly occurs when the study area is dominated by “spatial outliers” (i.e., high-low [HL]or low–high [LH] types) rather than extensive HH or LL clusters. In such cases, the positive and negative local associations cancel each other out at the global level, driving the global Moran’s I toward zero and rendering it statistically non-significant.

The results of this study correspond exactly to this scenario. As shown in Fig. 8 and Table 8, the spatial structure of carbon emissions in Hubei Province is characterized primarily by HL types (Wuhan, Xiangyang, Yichang: high emissions surrounded by low emissions) and LH types (Xianning, Ezhou, Suizhou: low emissions surrounded by high emissions), with no stable HH or LL clusters. Therefore, the identification and discussion of “riverside high-emission isolates” (HL type) represent valid findings based on local spatial autocorrelation (LISA) and do not contradict the non-significant global Moran’s I. On the contrary, it is precisely the existence of these “isolates” that explains why the global clustering pattern is not significant.

## Discussion

### Theoretical and practical implications of principal findings

Based on land use remote sensing data from 2000 to 2020 and statistical data from 2005 to 2020 for Hubei Province, this study systematically revealed the spatiotemporal evolution characteristics of land use carbon effects driven by NQPF, providing a scientific basis for regional low-carbon transition.

#### Dimensional characteristics of NQPF “Green Transformation”

The findings indicate that the NQPF index in Hubei Province increased from 0.152 in 2005 to 0.860 in 2020 (465.8% growth), with an average annual growth rate of 12.26%, exhibiting pronounced phase-specific evolutionary characteristics. Regarding dimensional weight contributions, the “new objects of labor” dimension received the highest combined weight of 0.5921, followed by the “new means of labor” dimension at 0.2187 and the “new laborers” dimension at 0.1892. This result corroborates the essential attribute of NQPF as being “green development-oriented,” indicating that industrial structure optimization, resource utilization efficiency improvement, and ecological environmental protection occupy dominant positions in NQPF development. Among individual indicators, the five with the highest weights were C32 (ratio of industrial wastewater to total wastewater discharge, 0.0855), C42 (patent grants per 10,000 persons, 0.0736), C41 (patent applications per 10,000 persons, 0.0665), C23 (value added of high-tech industries, 0.0646), and A32 (internal R&D expenditure, 0.0614), suggesting that the intensity of scientific and technological innovation and green ecological sustainability constitute the core elements of NQPF.

In terms of developmental stages, the province progressed through four phases: an initiation phase (2005–2007), a rapid growth phase (2007–2011, with an average annual growth rate of 20.0%), a stable growth phase (2011–2016, with an average annual growth rate of 5.32%), and an accelerated advancement phase (2016–2020, with an average annual growth rate of 13.78%). Two growth peaks occurred during 2007–2008 (36.7%) and 2017–2019 (17.4%–17.6%), corresponding to the policy-driven effects of the “resource-conserving and environmentally friendly society” pilot zone designation in 2007 and the accelerated emergence of strategic emerging industries, respectively. This phase-specific evolution aligns closely with major policy milestones in Hubei Province, indicating that policy drivers constitute a significant impetus for NQPF development.

#### The “Decoupling Intensification” pattern of land use carbon emissions

Tapio decoupling analysis revealed that all three periods (2005–2010, 2010–2015, 2015–2020) exhibited “weak decoupling” status; however, the decoupling elasticity coefficient displayed a continuous declining trend (0.740 → 0.588 → 0.261), indicating a strengthening constraining effect of NQPF on carbon emission growth. Notably, 2015–2020 represented a “high-intensity decoupling phase,” during which NQPF growth (64.25%) was 3.84 times that of carbon emission growth (16.74%), indicating improving development quality and a critical transitional stage toward strong decoupling.

The underlying mechanisms of this “decoupling intensification” pattern primarily stem from: first, the rapid emergence of strategic emerging industries, with increasing shares of low-carbon sectors such as optoelectronics and biopharmaceuticals; second, enhanced green technology innovation capacity; third, substantial improvements in energy utilization efficiency with continuous declines in carbon emission intensity per unit of GDP; and fourth, the deepening advancement of ecological civilization construction. Regarding construction land carbon emissions, emissions increased from 6909.06 × 104 t to 13,623.42 × 104 t between 2005 and 2010 (97.2% growth), whereas the growth rate decelerated markedly during 2015–2020, indicating that land use patterns are transitioning toward intensification and low-carbon modes. Nevertheless, despite continuous strengthening of decoupling intensity, none of the three periods achieved a breakthrough from “weak decoupling” to “strong decoupling” (ε < 0), indicating that Hubei Province must accelerate NQPF development to achieve absolute reductions in carbon emissions expeditiously.

#### The “Riverside Isolate” characteristics of carbon emissions spatial patterns

Moran’s I analysis revealed that Moran’s I values for all five years from 2000 to 2020 were proximate to the expected value E(I) =  − 0.0625 (specifically − 0.0425, − 0.0709, − 0.0748, − 0.0763, and − 0.0709), with deviation values failing to pass significance testing (|Z|< 1.96). This indicates that carbon emissions at the prefecture-level city scale exhibited no significant HH or LL clustering, displaying random distribution characteristics. Concerning temporal evolution, Moran’s I exhibited a U-shaped pattern, with negative correlation progressively strengthening from 2000 to 2015 (increasing spatial heterogeneity), followed by a slight increase from 2015 to 2020 (trending toward relative stabilization).

Local spatial autocorrelation analysis demonstrated that Wuhan and Xiangyang remained consistently positioned in the HL quadrant (high emission–low spatial lag) throughout 2000–2020, while Yichang became stably positioned in the HL quadrant from 2005, forming typical “isolated high-emission centers.” These riverside cities emerged as high-carbon isolates but did not transmit emissions to surrounding cities through industrial transfer or technology diffusion; instead, neighboring cities (Xianning, Ezhou, Suizhou, and others) formed low-carbon depressions (LH quadrant). This “isolate effect” reflects a distinctive spatial logic under the administratively-driven economic system: individual cities advance industrialization and urbanization through administrative means, lacking effective industrial coordination and spatial spillover effects between central and peripheral cities. This finding offers important implications for regional carbon reduction policies: policy formulation should emphasize “administratively-driven coordination mechanisms,” such as establishing cross-municipal carbon emission trading systems, creating regional emission reduction funds, and promoting coordinated emission reduction along industrial chains.

## Research limitations and future directions

This study selected five temporal nodes (2000, 2005, 2010, 2015, 2020) at five-year intervals, which cannot capture short-term annual fluctuations. Due to data availability constraints, municipal-level NQPF indices were not calculated, precluding spatial coupling analysis between NQPF and carbon emissions. Carbon emission accounting employed the coefficient method, with cultivated land carbon emission coefficients determined based on the ratio of total net carbon emissions to cultivated land area during 2000–2020, without accounting for inter-annual variations. Future research could employ annual data and determine more accurate carbon emission/sequestration coefficients for Hubei Province through field surveys and laboratory analyses.

Moran’s I analysis employed the inverse distance method to construct the spatial weight matrix, considering only geographic distance without accounting for factors such as economic linkages and transportation accessibility. The Tapio model assumes constant decoupling elasticity coefficients within time periods, whereas time-varying characteristics may exist in practice. Future research could conduct robustness tests using multiple weight matrices, including economic distance matrices and transportation distance matrices, and employ time-varying parameter models to dynamically estimate decoupling elasticity.

Future research directions may proceed along the following trajectories: first,deepening mechanistic research by employing mediation effect models and moderation effect models to analyze the transmission mechanisms through which NQPF influences carbon emissions; second, conducting scenario simulations by constructing system dynamics (SD) models to simulate carbon emission scenarios under different NQPF development pathways; third, undertaking cross-regional comparative studies by selecting eastern developed regions (Jiangsu, Zhejiang) and western less-developed regions (Gansu, Ningxia) for comparative analysis to explore the applicability of the Hubei experience; and fourth, investigating micro-level agent behavior by analyzing how NQPF influences production decisions and consumption behavior from enterprise and resident perspectives.

The findings of this study have direct policy relevance to the advancement of China’s ‘dual carbon’ strategy. First, the sustained strengthening of weak decoupling between NQPF and carbon emissions in Hubei Province supports the feasibility of achieving carbon reduction through improved economic development quality (rather than simply controlling growth rates) ^7^. Second, the finding that construction land is the dominant carbon source suggests that energy consumption structure adjustment should be prioritized in industrial and urban construction sectors ^9^. Third, if Hubei Province maintains its current NQPF development momentum and continues to strengthen decoupling intensity, it could provide a typical central China case study for achieving national carbon neutrality goals ^8^.

## Conclusions

Using Hubei Province as a case study and based on land use remote sensing data from 2000 to 2020 and statistical data from 2005 to 2020, this study systematically analyzed the spatiotemporal evolution characteristics and coupling relationships of land use carbon effects driven by NQPF. The principal conclusions are as follows:

1. Land use carbon emissions exhibited rapid growth characterized by transformation from “carbon sink to carbon source”. Net land use carbon emissions in Hubei Province increased from 4,118.38 × 104 t in 2000 to 19,867.15 × 104 t in 2020, representing a growth rate of 382.4% and completing a transformation from “carbon sink” to “carbon source.” Carbon emissions from construction land increased from 4,331.64 × 104 t to 20,095.02 × 104 t (363.9% growth), with its proportion of total carbon sources rising from 91.2% to 98.1%, constituting the dominant driver of carbon emission growth. Regarding spatial patterns, the combined carbon emissions of Wuhan, Xiangyang, and Yichang in 2020 totaled 10,755.21 × 104 t, accounting for 54.2% of the provincial total and forming a triangular high-emission configuration. Carbon emission growth exhibited a three-stage pattern characterized by “rapid growth–sustained growth–decelerated growth.”
2. NQPF exhibited sustained rapid growth with pronounced policy-driven characteristics. The NQPF index in Hubei Province increased from 0.152 in 2005 to 0.860 in 2020, representing a growth rate of 465.8% and an average annual growth rate of 12.26%. The developmental trajectory followed an evolutionary path characterized by “policy-driven initiation–fluctuating growth–accelerated advancement,” progressing through four stages: an initiation phase, a rapid growth phase (2007–2011, with an average annual growth rate of 20.0%), a stable growth phase, and an accelerated advancement phase (2016–2020, with an average annual growth rate of 13.78%). Regarding dimensional weights, “new objects of labor” accounted for 0.5921, “new means of labor” for 0.2187, and “new laborers” for 0.1892, indicating that green development and scientific and technological innovation constitute the core elements of NQPF.
3. NQPF and carbon emissions exhibited “weak decoupling” with progressively intensifying decoupling strength. Tapio decoupling analysis demonstrated that all three periods from 2005 to 2020 exhibited “weak decoupling” status, with the decoupling elasticity coefficient declining from 0.740 to 0.588 to 0.261, indicating a strengthening constraining effect of NQPF on carbon emission growth. The period 2015–2020 represented a “high-intensity decoupling phase,” during which NQPF growth (64.25%) was 3.84 times that of carbon emission growth (16.74%), indicating that economic growth patterns in Hubei Province are transitioning from high-carbon to low-carbon modes, with continuously improving green development quality.
4. Carbon emission spatial patterns exhibited “riverside isolate” characteristics without significant clustering. Moran’s I analysis demonstrated that carbon emissions from 2000 to 2020 exhibited no significant HH or LL clustering spatially (Moran’s I proximate to the expected value of − 0.0625, |Z|< 1.96), displaying random distribution characteristics. Local spatial autocorrelation analysis revealed that riverside cities including Wuhan, Xiangyang, and Yichang remained consistently positioned in the HL quadrant, forming “isolated high-emission centers”; Jingmen, Jingzhou, Qianjiang, Shiyan, and Tianmen formed a stable low-carbon cluster (LL quadrant); and Xianning, Ezhou, Suizhou, and others formed low-carbon depressions (LH quadrant). Spatial heterogeneity was significant but clustering was not pronounced, with carbon emissions in individual cities primarily driven by their own development patterns and limited spatial spillover effects.

This study represents the first integration of NQPF theory into land use carbon effect research, constructing an analytical framework of “NQPF–land use–carbon emissions” that provides a scientific basis for regional low-carbon transition and high-quality development. The findings regarding “weak decoupling” status, “spatial non-clustering” characteristics, and “riverside isolate” patterns enrich theoretical understanding of NQPF and land use carbon emissions, offering empirical reference for low-carbon development in central China and nationwide.

## Data Availability

The original contributions presented in this study are included in the article. Data will be made available on request.
